# In-hospital outcomes following sleeve gastrectomy and Roux-en-Y gastric bypass: a real-world multicenter study in Germany

**DOI:** 10.1007/s00423-026-04064-9

**Published:** 2026-04-30

**Authors:** Sarah Krieg, Claus F. Eisenberger, Andreas Krieg, Karel Kostev

**Affiliations:** 1 Medical School and University Medical Center OWL, Mara Hospital, University Clinic for People with Neurodevelopmental Disorders, Bielefeld University, Maraweg 21, 33617 Bielefeld, Germany; 2 Department of Abdominal, Tumor, Transplant and Vascular Surgery, Cologne-Merheim Medical Center, Witten/Herdecke University, Ostmerheimer Str. 200, 51109 Cologne, Germany; 3 Department of General and Visceral Surgery, Thoracic Surgery and Proctology, Medical Campus OWL, University Hospital Herford, Ruhr University Bochum, Schwarzenmoorstr. 70, 32049 Herford, Germany; 4 Epidemiology, IQVIA, Frankfurt, 60549 Germany

**Keywords:** Metabolic bariatric surgery, Sleeve gastrectomy, Roux-en-Y gastric bypass, Obesity

## Abstract

**Purpose:**

Metabolic bariatric surgery (MBS) is an effective treatment for severe obesity. While randomized trials have shown comparable medium-term outcomes for laparoscopic sleeve gastrectomy (LSG) and Roux-en-Y gastric bypass (LRYGB), real-world data on perioperative safety remain limited. This study compares in-hospital mortality, complications, and length of stay between LSG and LRYGB in a large German cohort.

**Methods:**

We performed a cross-sectional analysis of anonymized hospital data from 49 German institutions (January 2019–January 2024), including 3,389 MBS procedures (LSG: *n* = 2,664; LRYGB: *n* = 725). Outcomes included in-hospital mortality, defined perioperative complications (e.g., hemorrhage, infection, anastomotic leak), and length of hospital stay. Logistic regression adjusted for age, sex, BMI categories, and comorbidities was used to assess associations.

**Results:**

Most individuals (69.1%) had a BMI ≥ 40 kg/m². LSG was more common in individuals with BMI ≥ 50 kg/m², whereas LRYGB was preferred in individuals with lower BMI and higher metabolic burden. In-hospital mortality was low: two deaths occurred after LRYGB (0.3%), none after LSG. Overall complication rates were similar (LSG: 3.9%; LRYGB: 4.1%). We did not observe statistically significant differences in complication risk; however, confidence intervals were wide, reflecting limited statistical power. Median length of hospital stay was three days for both groups; prolonged stays (> 7 days) occurred in 3.5% (LSG) vs. 3.6% (LRYGB; *p* = 0.863).

**Conclusion:**

Both procedures demonstrated low complication and mortality rates in routine clinical practice.

## Introduction

The global rise in obesity remains a major public health concern [1]. Since 1975, its prevalence has nearly tripled worldwide. In high-income countries, a substantial proportion of the adult population is affected, with current estimates indicating that approximately 25% of German adults live with obesity [2, 3]. Obesity is associated with a significantly increased risk of numerous chronic diseases—including type 2 diabetes mellitus, hypertension, dyslipidemia, cardiovascular disease, and certain cancers [4–7] — and is linked to reduced life expectancy [8]. A considerable proportion of this disease burden can be attributed to individuals with a BMI ≥ 50 kg/m², who are characterized by pronounced metabolic stress and increased perioperative challenges [9].

The treatment of obesity is approached through a multimodal strategy, integrating nutrition, exercise, and behavioral therapy [10]. In addition, novel obesity management medications (OMMs), including GLP-1 receptor agonists and dual incretin mimetics, have exhibited promising results in clinical trials for weight reduction and glycemic control [11, 12]. However, in individuals with severe obesity, surgical interventions are currently regarded as the most efficacious approach for long-term weight reduction and improvement of associated medical problems [13, 14]. Long-term data sets, including those from the Swedish Obese Subjects (SOS) study, have demonstrated a significant decrease in type 2 diabetes mellitus, cardiovascular incidents, and overall mortality [15, 16].

The current German guideline advocates metabolic and bariatric surgery (MBS) for individuals with a BMI ≥ 40 kg/m², or ≥ 35 kg/m² with associated medical problems, if structured conservative therapy has failed [17]. However, in light of an international paradigm shift, MBS procedures are increasingly recognized as having independent metabolic efficacy [14, 18–21]. Consequently, these procedures are now also referred to as metabolic surgery. The mechanisms are considered multifactorial, involving changes in incretin secretion, bile acid metabolism, intestinal glucose utilization, and gut microbiota [21, 22].

In addition, recent international guidelines have broadened the indications for metabolic surgery. The 2022 joint statement by the ASMBS and IFSO recommends surgery for patients with a BMI ≥ 35 kg/m² irrespective of comorbidities, and for those with a BMI 30–34.9 kg/m² in the presence of metabolic disease such as type 2 diabetes [23]. These updated thresholds highlight the metabolic benefits of surgery beyond weight reduction and support earlier, risk-adapted intervention.

The most frequently performed MBS procedures worldwide are laparoscopic sleeve gastrectomy (LSG) and Roux-en-Y gastric bypass (LRYGB) [14]. LRYGB involves creating a gastric pouch and bypassing the proximal small intestine, while LSG entails a vertical gastrectomy with fundus resection—affecting appetite regulation through hormonal changes [14, 24].

Randomized studies, including SM-BOSS and SLEEVEPASS, have demonstrated comparable outcomes regarding medium-term weight loss in individuals with BMI 35–50 kg/m² [24, 25]. LRYGB appears more effective for remission of obesity-associated comorbidities, particularly type 2 diabetes mellitus, as shown in registry data and subgroup analyses [19, 26]. There are known procedure-specific risks: LSG is associated with gastroesophageal reflux [27], while LRYGB may result in micronutrient deficiencies, especially of iron, vitamin B12, and fat-soluble vitamins. In addition, conventional ERCP is not feasible after LRYGB [28, 29].

However, perioperative complication rates of both procedures are inconsistently reported in the literature and vary by patient population, study design, and care structure [26, 30].

The BEST study, one of the largest randomized European trials, compared LSG and LRYGB under standardized conditions. It included individuals with BMI 35–50 kg/m² across 23 Scandinavian centers [31]. Although the study had strong internal validity, its design may limit generalizability to broader real-world populations. Real-world data are essential to guide clinical practice and evaluate outcomes of MBS—particularly given the rising use and high cost of OMMs, which may hinder long-term adherence [32, 33].

Against this background, the present study aims to analyze in-hospital mortality, complications, and length of stay after LSG and LRYGB under routine clinical conditions. Unlike previous randomized or registry-based studies predominantly conducted in specialized high-volume centers, this multicenter analysis includes hospitals of different care levels across Germany, thereby providing real-world evidence on the safety and utilization of metabolic and bariatric surgery in everyday clinical practice.

## Materials and methods

### Data source

**Fig. 1:**
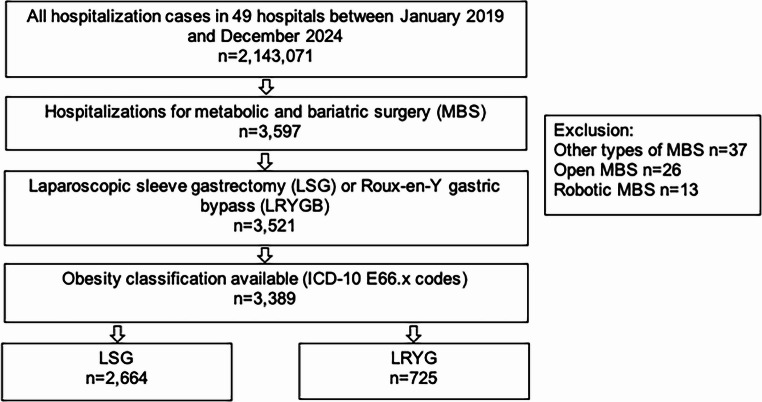
Selection of study individuals

This multicenter cross-sectional study was based on anonymized data from the IQVIA hospital database, covering 49 hospitals across Germany and totaling 2,143,071 inpatient cases between January 1, 2019, and January 31, 2024. The participating hospitals included primary care centers, standard and maximum care institutions, and university hospitals. Metabolic and bariatric procedures were performed in nine of these hospitals. Data were collected in accordance with § 21 of the Hospital Compensation Act (KHEntgG) and transmitted to the Institute for the Hospital Remuneration System (InEK). All patient and case identifiers were anonymized prior to data export (Fig. 1).

## Study population

The study population consisted of adult inpatients (aged ≥ 18 years) who underwent primary metabolic and bariatric surgery (MBS) during the observation period. Our aim was to include only primary metabolic and bariatric surgeries. Cases with ICD or OPS codes indicating revisional surgeries were excluded. Only procedures classified as either laparoscopic sleeve gastrectomy (LSG) or laparoscopic Roux-en-Y gastric bypass (LRYGB) were included. Cases involving other types of MBS (*n* = 37), open surgeries (*n* = 26), or robot-assisted procedures (*n* = 13) were infrequent and excluded from the analysis.

### Study outcome

The primary outcomes included in-hospital mortality, documented perioperative complications, and length of hospital stay. Complications were identified using ICD-10 codes and restricted to directly surgery-related events such as hemorrhage or hematoma (T81.0), accidental puncture or laceration (T81.2), wound disruption (T81.3), infections (T81.4, T82.7), and anastomotic leaks or suture failure (K91.83).

### Statistical analyses

Baseline characteristics included age (≤ 30, 31–40, 41–50, > 50 years), sex, BMI categories, and comorbid conditions documented in ≥ 5% of the study population—such as thyroid disorders (E00–E06), diabetes mellitus (E10–E14), dyslipidemia (E78), any heart disease (I20–I52), and sleep disorders (G47). Group comparisons between LSG and LRYGB were conducted using t-tests (for continuous variables) and chi-square tests (for categorical variables). Multivariable logistic regression models were employed to evaluate associations between procedure type (LRYGB vs. LSG) and clinical outcomes, adjusted for age, sex, BMI categories, and comorbidities. Results are reported as adjusted odds ratios (ORs) with 95% confidence intervals (CIs). Median hospital stay was compared, and the proportion of patients with prolonged hospitalization—defined as a stay of more than 7 days—was analyzed. A p-value < 0.05 was considered statistically significant. Statistical significance was set at *p* < 0.05. Analyses were conducted using SAS 9.4 (SAS Institute, Cary, USA).

## Results

### Selection of study individuals

A total of 3,389 inpatients underwent MBS during the study period, including 2,664 individuals undergoing LSG and 725undergoing LRYGB. The majority of individuals (69.1%) had a BMI ≥ 40 kg/m². LRYGB recipients were slightly older on average (44.3 vs. 42.4 years), more likely to be female (75.5% vs. 68.5%), and had a higher prevalence of BMI < 50 kg/m² and diabetes mellitus. Individuals undergoing LSG showed a higher prevalence of BMI ≥ 50 kg/m² and dyslipidemia (Table 1).

**Table 1:** Baseline characteristics of the study sample

	LSG(*n* = 2,664)	LRYGB(*n* = 725)	*P*-value
Age			
Mean age (SD)	42.4 (11.9)	44.3 (11.1)	
≤ 30 years, n (%)	481 (18.1)	81 (11.2)	< 0.001
31–40 years, n (%)	760 (28.5)	200 (27.6)	
41–50 years, n (%)	678 (23.4)	201 (29.0)	
> 50 years, n (%)	745 (28.0)	234 (32.2)	
Sex, n (%)			
Female	1,826 (68.5)	547 (75.5)	< 0.001
Male	838 (31.5)	178 (24.5)	
Documented diagnoses, n (%)			
Obesity I (30–34.9 kg/m²)	132 (4.9)	92 (12.7)	< 0.001
Obesity II (35–39.9 kg/m²)	624 (23.4)	199 (27.5)	0.025
Obesity III (40–49.9 kg/m²)	863 (32.4)	310 (42.8)	< 0.001
Obesity IV (50–59.9 kg/m²)	838 (31.5)	109 (15.0)	< 0.001
Obesity V (60 + kg/m²)	207 (7.8)	15 (2.1)	< 0.001
Thyroid gland disorder	701 (26.3)	165 (22.8)	0.052
Diabetes mellitus	788 (29.6)	266 (36.7)	< 0.001
Dyslipidemia	711 (26.7)	147 (20.3)	< 0.001
Hypertension	1,321 (49.6)	354 (48.8)	0.717
Sleep disorder	518 (19.4)	122 (16.8)	0.111
Any heart disease	144 (5.4)	43 (5.9)	0.583

### In-hospital mortality and complications

In-hospital mortality was low in both groups, with two deaths reported following LRYGB (0.3%) and none after LSG. The overall complication rates did not differ significantly between the groups (LSG: 3.9%; LRYGB: 4.1%). Hemorrhage and hematoma represented the most common complications (LSG: 3.0%; LRYGB: 1.8%). Anastomotic leakage or suture failure was observed in 0.8% of LSG cases and 1.5% of LRYGB cases. In multivariable logistic regression analysis, LRYGB was not associated with an increased risk of any complication (OR: 1.27; 95% CI: 0.82–1.97, although the confidence interval includes effect sizes that may be clinically relevant), nor with an increased risk of any specific complication (Table 2).

**Table 2:** Association of LRYGB as compared to LSG and in-hospital mortality and complications (multivariable logistic regression)

Outcome	Prevalence among individuals with LSG (*N*, %)	Prevalence among individuals with LRYGB (*N*, %)	OR (95% CI) *	*P* value
In-hospital mortality	0 (0.0)	2 (0.3)	-	
Any in-hospital complication	103 (3.9)	30 (4.1)	1.27 (0.82–1.97)	0.281
Hemorrhage or hematoma	79 (3.0)	13 (1.8)	0.74 (0.40–1.37)	0.334
Accidental puncture and laceration	0 (0.0)	4 (0.6)	-	
Wound disruption	4 (0.2)	3 (0.4)	1.50 (0.29–7.70)	0.628
Infection	12 (0.5)	14 (0.6)	1.36 (0.37–4.97)	0.638
Anastomotic leak or suture failure	21 (0.8)	11 (1.5)	1.70 (0.79–3.67)	0.177

*Multivariable logistic regression adjusted for age, sex, BMI categories, and diagnoses; missing OR means that event occurred in only one group and no calculation of OR was possible

### Length of hospital stay

The median length of stay was three days for both groups (interquartile range: one day). Prolonged hospitalizations (> 7 days) occurred in 3.5% of LSG cases and 3.6% of LRYGB cases (*p* = 0.863).

## Discussion

This multicenter analysis of 3,389 inpatient MBS in Germany, including 2,664 LSG and 725 LRYGB, offers a comprehensive overview of procedural utilization, safety, and complication profiles under real-world conditions. By complementing randomized controlled trial data with population-level routine care evidence, this study provides important context on clinical practice in the landscape of MBS in Germany. The predominance of LSG (approximately 79%) aligns with global procedural trends, as reported in the 2023 IFSO Global Registry (63.3% LSG vs. 28.8% LRYGB) [34]. This concordance reinforces the external validity of the findings and suggests that the observed procedural patterns may be extrapolated to similar healthcare systems.

Physiologically and technically, the two procedures differ substantially in their mechanisms of action and clinical implications. LSG involves vertical resection of the greater curvature of the stomach, including the fundus, which reduces gastric volume and significantly lowers circulating levels of ghrelin—the orexigenic hormone primarily produced in the gastric fundus. This hormonal change is associated with decreased appetite and altered hunger perception, contributing to weight loss beyond the effect of volume reduction alone [35, 36]. Moreover, LSG influences gastric emptying and may modulate enteroendocrine signaling, including GLP-1 and PYY secretion, though to a lesser extent than LRYGB. In contrast, LRYGB involves the creation of a small gastric pouch and a Roux-en-Y reconstruction of the small intestine, bypassing the proximal duodenum and jejunum [37]. This results not only in reduced caloric intake and nutrient absorption but also in significant changes in the gut hormonal milieu. Postprandial levels of incretins such as GLP-1 and PYY are markedly increased, which enhances satiety, improves insulin secretion, and contributes to the resolution of type 2 diabetes mellitus [38].

In the present cohort, individuals with a BMI ≥ 50 kg/m² were significantly more likely to undergo LSG than LRYGB (*p* < 0.001), with this trend becoming increasingly pronounced at higher BMI categories (BMI 50–59.9 kg/m²: 31.5% vs. 15.0%; BMI ≥ 60 kg/m²: 7.8% vs. 2.1%). This pattern is consistent with current clinical practice and likely reflects both the perceived perioperative risks in individuals with severe obesity and the strategic implementation of stepwise treatment algorithms—such as the ‘sleeve-then-bypass’ approach—adopted to reduce surgical risk and enable staged metabolic optimization [39].

Although neither procedure demonstrates absolute superiority, specific advantages have emerged based on patient characteristics. LRYGB has shown more pronounced metabolic effects in individuals with advanced type 2 diabetes mellitus or dyslipidemia. For instance, Bhandari et al. observed greater metabolic improvement following LRYGB in individuals with BMI ≥ 50 kg/m², despite comparable total weight loss across procedures [40].

Conversely, LSG appears preferable in individuals with a BMI ≥ 50 kg/m² due to its favorable perioperative safety profile. Howell et al. reported a complication rate of 5.3% in individuals with BMI ≥ 60 kg/m², with no significant difference between LSG and LRYGB, although a numerical trend favored LSG [41]. Hassan et al. confirmed significantly lower 30-day complication rates with LSG in a cohort of 18,861 individuals with BMI ≥ 60 kg/m² (2.2% vs. 4.0%; *p* = 0.048) [42]. Similarly, Wilkinson et al. found that LSG was associated with fewer reintubations, prolonged ventilation, ICU admissions, and septic events in a high-risk cohort [43].

These data support the view that LSG may be associated with lower perioperative morbidity in individuals with very high BMI. Reduced technical complexity and the absence of anastomoses may underlie this safety profile. The results emphasize the importance of individualized, risk-adapted procedural selection.

Procedure distribution in the present cohort also reflects differential use by metabolic status. LRYGB was more frequently performed among individuals with BMI 30–49.9 kg/m² and among those with diabetes mellitus (36.7% vs. 29.6%; *p* < 0.001), consistent with guideline recommendations favoring MBS in individuals with moderate obesity and associated medical problems [18].

This is further supported by updated ASMBS and IFSO guidelines, which recommend MBS for individuals with a BMI ≥ 35 kg/m² regardless of associated medical problems, and for those with a BMI of 30–34.9 kg/m² if such conditions are present [14]. These developments underscore a shift toward indications informed by metabolic and obesity-associated diseases. Interestingly, a higher prevalence of dyslipidemia was observed among individuals undergoing LSG (26.7% vs. 20.3%; *p* < 0.001), which may reflect the greater BMI burden in this subgroup, as the risk of dyslipidemia increases with BMI [44]. In such cases, LSG may be preferred despite the presence of metabolic and obesity-related conditions, due to its more favorable perioperative risk profile.

Gender-related trends were also observed, with more female individuals undergoing LRYGB (75.5% vs. 68.5%; *p* < 0.001). Similar findings were reported by Bal et al., who attributed this to a greater prevalence of body image concerns and health-seeking behavior among women [45].

A notable finding was the high proportion of individuals with BMI ≥ 50 kg/m² (39.3% LSG; 17.1% LRYGB), suggesting that MBS in Germany is often initiated at advanced stages of obesity. In comparison with international registries (e.g., MBSAQIP), this points to potential delays in access to MBS. Contributing factors may include restrictive reimbursement policies, inconsistent referral practices, and regional disparities. These findings should inform discussions on indication criteria and access to care in Germany [14, 18].

Regarding perioperative outcomes—one of the central findings of this study—both procedures were associated with low complication rates: 3.9% for LSG and 4.1% for LRYGB. The most commonly reported events were bleeding and hematomas. Anastomotic leaks or suture failures occurred in 0.8% of LSG cases and 1.5% of LRYGB cases. In-hospital mortality was very low, with no deaths following LSG and a mortality rate of 0.3% after LRYGB. These findings suggest low overall rates of coding-based perioperative complications in both groups. However, given the limited statistical power, the absence of statistically significant differences should not be interpreted as evidence of equivalent safety. The observed rates are broadly consistent with international benchmarks, including those reported by the MBSAQIP program [46].

Importantly, these results are consistent with the SM-BOSS and SLEEVEPASS randomized trials, which reported no significant differences in complication rates [24, 25]. The low morbidity observed may reflect the growing implementation of standardized protocols and structural quality measures, even in the absence of formalized programs like MBSAQIP in Germany.

Further confirmation comes from the BEST trial, a randomized, multicenter study involving 1,735 individuals (BMI 35–50 kg/m²) [31]. Complication rates were low and did not differ significantly between groups (4.6% vs. 6.3%; *p* = 0.11), including serious complications (1.7% vs. 2.7%) and 90-day mortality (0% in both arms) [31]. Notably, LSG was associated with a shorter operative time (47 vs. 68 min), a finding with practical relevance for resource allocation. In contrast to BEST, the present study included individuals with BMI > 50 kg/m² and drew from a diverse sample of nine hospitals across Germany, enhancing generalizability and applicability to routine clinical settings.

A recent meta-analysis by Bregion et al., analyzing 28 studies with 156,767 individuals with BMI ≥ 50 kg/m², found no differences in mortality (OR 1.28; *p* = 0.311) or complication rates (OR 1.22; *p* = 0.287) between procedures [47]. LSG was associated with shorter operative time, length of hospital stay, and fewer conversions to open surgery [47]. However, methodological limitations (e.g., retrospective design, heterogeneity, unadjusted confounders) limit the strength of inference. Notably, the present study includes a broader BMI spectrum and captures care under real-world conditions. Collectively, these findings reinforce the need for individualized metabolic and bariatric treatment strategies that consider obesity-related disease profiles, procedural risk, and long-term treatment goals as part of a shared decision-making process.

Several limitations inherent to the use of administrative data must be considered. The data were originally collected for reimbursement purposes and therefore lack detailed clinical information. Although individuals could be grouped into predefined BMI categories, individual-level BMI values were not available for more granular analyses. As a result, BMI could only be included in categorical form rather than as a continuous variable. Furthermore, important clinical variables such as ASA class, functional status, smoking status, and previous abdominal surgery were not available, limiting the ability to adjust for confounding.

Additionally, variables such as the severity of obesity-related conditions, operative duration, intraoperative findings, and whether procedures were primary or revisional could not be assessed. The accuracy of ICD-10 coding varies; therefore, analyses based on administrative data should be interpreted as reflecting coding-based events rather than clinically adjudicated outcomes. Perioperative complications were identified through ICD-10 codes, which do not allow classification by severity using systems such as Clavien-Dindo. In this context, it is important to note that because conversions to open surgery are coded as open procedures, they may have been excluded, potentially underestimating complication rates. In addition, the low number of events limits statistical power, and the study was not designed to establish equivalence between procedures. Functional outcomes—including postoperative gastroesophageal reflux, dumping syndrome, and micronutrient deficiencies—were not captured.

Although events such as pulmonary embolism, myocardial infarction, renal failure, or sepsis may occur during or after surgery, the administrative dataset does not provide timing information that would allow us to distinguish new postoperative events from pre-existing conditions present on admission. Many individuals undergoing MBS have chronic cardiometabolic or renal disease at baseline, and including these ICD-10 codes would risk misclassifying pre-existing conditions as complications. Therefore, we restricted the definition of complications to ICD-10 codes (e.g., T81.x, K91.83) that unambiguously represent intra- or postoperative surgical complications.

Furthermore, the analysis was confined to the inpatient stay, which precludes conclusions regarding long-term outcomes such as weight maintenance, health-related quality of life, and the need for reoperation. Lastly, information on institutional characteristics—such as surgical experience, hospital volume, or participation in structured quality improvement programs—was not available, limiting the ability to contextualize the findings.

Nevertheless, this study is strengthened by its large and heterogeneous dataset, encompassing 3,389 procedures from nine hospitals across Germany. The inclusion of individuals across a broad BMI spectrum—including both moderate and severe obesity—enhances the generalizability of the findings and reflects the complexity of routine clinical care. Moreover, the alignment of these findings with international trial and registry data supports the robustness and external validity of the observed trends in perioperative outcomes and procedural distribution.

 In conclusion, this nationwide study provides robust real-world evidence on the safety and utilization patterns of LSG and LRYGB in Germany. Both procedures were associated with low perioperative complication rates and very low in-hospital mortality in routine clinical practice. LSG was more frequently performed in individuals with severe obesity (BMI ≥ 50 kg/m²), while LRYGB was favored among those with pronounced metabolic disease at lower BMI levels. The high proportion of individuals with BMI ≥ 50 kg/m² suggests that MBS in Germany is often initiated at an advanced point along the BMI spectrum. Earlier surgical referral, especially for metabolically high-risk individuals, may improve outcomes and broaden access.

While no statistically significant differences in complication rates were observed between LSG and LRYGB, the study was not powered to exclude clinically relevant differences, and comparative safety between procedures cannot be conclusively determined. Procedure selection should therefore continue to be guided by individual patient characteristics, including BMI class, metabolic profile, long-term goals, and patient preference.

## Data Availability

The datasets analyzed during the current study are not publicly available due to data protection regulations but are available from the corresponding author upon reasonable request and with permission from the data provider.

## References

[1] Okunogbe A, Nugent R, Spencer G, Powis J, Ralston J, Wilding J (2022) Economic impacts of overweight and obesity: current and future estimates for 161 countries. BMJ Glob Health 7 10.1136/bmjgh-2022-009773 10.1136/bmjgh-2022-009773 PMC9494015 36130777

[2] Collaboration NCDRF (2017) Worldwide trends in body-mass index, underweight, overweight, and obesity from 1975 to 2016: a pooled analysis of 2416 population-based measurement studies in 128.9 million children, adolescents, and adults. Lancet 390:2627–2642. 10.1016/S0140-6736(17)32129-3 29029897 10.1016/S0140-6736(17)32129-3 PMC5735219

[3] Starker A, Schienkiewitz A, Damerow S, Kuhnert R (2025) Prevalence of obesity and smoking among adults in Germany - trends from 2003 to 2023. J Health Monit 10:e13038. 10.25646/13038 40196747 10.25646/13038 PMC11973604

[4] Abdullah A, Peeters A, de Courten M, Stoelwinder J (2010) The magnitude of association between overweight and obesity and the risk of diabetes: a meta-analysis of prospective cohort studies. Diabetes Res Clin Pract 89:309–319. 10.1016/j.diabres.2010.04.012 20493574 10.1016/j.diabres.2010.04.012

[5] Renehan AG, Tyson M, Egger M, Heller RF, Zwahlen M (2008) Body-mass index and incidence of cancer: a systematic review and meta-analysis of prospective observational studies. Lancet 371:569–578. 10.1016/S0140-6736(08)60269-X 18280327 10.1016/S0140-6736(08)60269-X

[6] Felson DT, Anderson JJ, Naimark A, Walker AM, Meenan RF (1988) Obesity and knee osteoarthritis. The Framingham Study. Ann Intern Med 109:18–24. 10.7326/0003-4819-109-1-18 3377350 10.7326/0003-4819-109-1-18

[7] Luppino FS, de Wit LM, Bouvy PF, Stijnen T, Cuijpers P, Penninx BW, Zitman FG (2010) Overweight, obesity, and depression: a systematic review and meta-analysis of longitudinal studies. Arch Gen Psychiatry 67:220–229. 10.1001/archgenpsychiatry.2010.2 20194822 10.1001/archgenpsychiatry.2010.2

[8] Prospective Studies C, Whitlock G, Lewington S, Sherliker P, Clarke R, Emberson J, Halsey J, Qizilbash N, Collins R, Peto R (2009) Body-mass index and cause-specific mortality in 900 000 adults: collaborative analyses of 57 prospective studies. Lancet 373:1083–1096. 10.1016/S0140-6736(09)60318-4 19299006 10.1016/S0140-6736(09)60318-4 PMC2662372

[9] Verhoeff K, Mocanu V, Dang J, Purich K, Switzer NJ, Birch DW, Karmali S (2022) Five Years of MBSAQIP Data: Characteristics, Outcomes, and Trends for Patients with Super-obesity. Obes Surg 32:406–415. 10.1007/s11695-021-05786-z 34782985 10.1007/s11695-021-05786-z

[10] Yumuk V, Tsigos C, Fried M, Schindler K, Busetto L, Micic D, Toplak H, Obesity Management Task Force of the European Association for the Study of O (2015) European Guidelines for Obesity Management in Adults. Obes Facts 8:402–424. 10.1159/000442721 26641646 10.1159/000442721 PMC5644856

[11] Wilding JPH, Batterham RL, Calanna S, Davies M, Van Gaal LF, Lingvay I, McGowan BM, Rosenstock J, Tran MTD, Wadden TA, Wharton S, Yokote K, Zeuthen N, Kushner RF, Group SS (2021) Once-Weekly Semaglutide in Adults with Overweight or Obesity. N Engl J Med 384:989–1002. 10.1056/NEJMoa2032183 33567185 10.1056/NEJMoa2032183

[12] Jastreboff AM, Aronne LJ, Ahmad NN, Wharton S, Connery L, Alves B, Kiyosue A, Zhang S, Liu B, Bunck MC, Stefanski A, Investigators S- (2022) Tirzepatide Once Weekly for the Treatment of Obesity. N Engl J Med 387:205–216. 10.1056/NEJMoa2206038 35658024 10.1056/NEJMoa2206038

[13] Courcoulas AP, Christian NJ, Belle SH, Berk PD, Flum DR, Garcia L, Horlick M, Kalarchian MA, King WC, Mitchell JE, Patterson EJ, Pender JR, Pomp A, Pories WJ, Thirlby RC, Yanovski SZ, Wolfe BM, Longitudinal Assessment of Bariatric Surgery C (2013) Weight change and health outcomes at 3 years after bariatric surgery among individuals with severe obesity. JAMA 310:2416–2425. 10.1001/jama.2013.280928 24189773 10.1001/jama.2013.280928 PMC3955952

[14] Hsu JL, Farrell TM (2024) Updates in Bariatric Surgery. Am Surg 90:925–933. 10.1177/00031348231220576 38060198 10.1177/00031348231220576

[15] Sjostrom L (2013) Review of the key results from the Swedish Obese Subjects (SOS) trial - a prospective controlled intervention study of bariatric surgery. J Intern Med 273:219–234. 10.1111/joim.12012 23163728 10.1111/joim.12012

[16] Sjostrom L, Peltonen M, Jacobson P, Sjostrom CD, Karason K, Wedel H, Ahlin S, Anveden A, Bengtsson C, Bergmark G, Bouchard C, Carlsson B, Dahlgren S, Karlsson J, Lindroos AK, Lonroth H, Narbro K, Naslund I, Olbers T, Svensson PA, Carlsson LM (2012) Bariatric surgery and long-term cardiovascular events. JAMA 307:56–65. 10.1001/jama.2011.1914 22215166 10.1001/jama.2011.1914

[17] Deutsche Adipositas-Gesellschaft (DAG) e.V. S3-Leitlinie Adipositas - prävention und therapie. Version 5.0 Oktober 2024 https://register.awmf.org/de/leitlinien/detail/050-001. Accessed on 7/6/2025

[18] Rubino F, Nathan DM, Eckel RH, Schauer PR, Alberti KG, Zimmet PZ, Del Prato S, Ji L, Sadikot SM, Herman WH, Amiel SA, Kaplan LM, Taroncher-Oldenburg G, Cummings DE (2016) Metabolic Surgery in the Treatment Algorithm for Type 2 Diabetes: A Joint Statement by International Diabetes Organizations. Diabetes Care 39:861–877. 10.2337/dc16-0236 27222544 10.2337/dc16-0236

[19] Mingrone G, Panunzi S, De Gaetano A, Guidone C, Iaconelli A, Capristo E, Chamseddine G, Bornstein SR, Rubino F (2021) Metabolic surgery versus conventional medical therapy in patients with type 2 diabetes: 10-year follow-up of an open-label, single-centre, randomised controlled trial. Lancet 397:293–304. 10.1016/S0140-6736(20)32649-0 33485454 10.1016/S0140-6736(20)32649-0

[20] Schauer PR, Kashyap SR, Wolski K, Brethauer SA, Kirwan JP, Pothier CE, Thomas S, Abood B, Nissen SE, Bhatt DL (2012) Bariatric surgery versus intensive medical therapy in obese patients with diabetes. N Engl J Med 366:1567–1576. 10.1056/NEJMoa1200225 22449319 10.1056/NEJMoa1200225 PMC3372918

[21] Ji Y, Lee H, Kaura S, Yip J, Sun H, Guan L, Han W, Ding Y (2021) Effect of bariatric surgery on metabolic diseases and underlying mechanisms. Biomolecules 11 10.3390/biom11111582 10.3390/biom11111582 PMC8615605 34827579

[22] Kaska L, Sledzinski T, Chomiczewska A, Dettlaff-Pokora A, Swierczynski J (2016) Improved glucose metabolism following bariatric surgery is associated with increased circulating bile acid concentrations and remodeling of the gut microbiome. World J Gastroenterol 22:8698–8719. 10.3748/wjg.v22.i39.8698 27818587 10.3748/wjg.v22.i39.8698 PMC5075546

[23] Eisenberg D, Shikora SA, Aarts E, Aminian A, Angrisani L, Cohen RV, De Luca M, Faria SL, Goodpaster KPS, Haddad A, Himpens JM, Kow L, Kurian M, Loi K, Mahawar K, Nimeri A, O’Kane M, Papasavas PK, Ponce J, Pratt JSA, Rogers AM, Steele KE, Suter M, Kothari SN (2022) 2022 American Society for Metabolic and Bariatric Surgery (ASMBS) and International Federation for the Surgery of Obesity and Metabolic Disorders (IFSO): Indications for Metabolic and Bariatric Surgery. Surg Obes Relat Dis 18:1345–1356. 10.1016/j.soard.2022.08.013 36280539 10.1016/j.soard.2022.08.013

[24] Peterli R, Wolnerhanssen BK, Peters T, Vetter D, Kroll D, Borbely Y, Schultes B, Beglinger C, Drewe J, Schiesser M, Nett P, Bueter M (2018) Effect of Laparoscopic Sleeve Gastrectomy vs Laparoscopic Roux-en-Y Gastric Bypass on Weight Loss in Patients With Morbid Obesity: The SM-BOSS Randomized Clinical Trial. JAMA 319:255–265. 10.1001/jama.2017.20897 29340679 10.1001/jama.2017.20897 PMC5833546

[25] Salminen P, Gronroos S, Helmio M, Hurme S, Juuti A, Juusela R, Peromaa-Haavisto P, Leivonen M, Nuutila P, Ovaska J (2022) Effect of Laparoscopic Sleeve Gastrectomy vs Roux-en-Y Gastric Bypass on Weight Loss, Comorbidities, and Reflux at 10 Years in Adult Patients With Obesity: The SLEEVEPASS Randomized Clinical Trial. JAMA Surg 157:656–666. 10.1001/jamasurg.2022.2229 35731535 10.1001/jamasurg.2022.2229 PMC9218929

[26] Wiggins T, Guidozzi N, Welbourn R, Ahmed AR, Markar SR (2020) Association of bariatric surgery with all-cause mortality and incidence of obesity-related disease at a population level: A systematic review and meta-analysis. PLoS Med 17:e1003206. 10.1371/journal.pmed.1003206 32722673 10.1371/journal.pmed.1003206 PMC7386646

[27] Melissas J, Braghetto I, Molina JC, Silecchia G, Iossa A, Iannelli A, Foletto M (2015) Gastroesophageal Reflux Disease and Sleeve Gastrectomy. Obes Surg 25:2430–2435. 10.1007/s11695-015-1906-1 26428250 10.1007/s11695-015-1906-1

[28] Bloomberg RD, Fleishman A, Nalle JE, Herron DM, Kini S (2005) Nutritional deficiencies following bariatric surgery: what have we learned? Obes Surg 15:145–154. 10.1381/0960892053268264 15802055 10.1381/0960892053268264

[29] Moreels TG (2019) Endoscopic retrograde cholangiopancreatography in Roux-en-Y gastric bypass patients. Minerva Chir 74:326–333. 10.23736/S0026-4733.18.07929-4 30334397 10.23736/S0026-4733.18.07929-4

[30] Clapp B, Lee I, Liggett E, Cutshall M, Tudor B, Pradhan G, Aguirre K, Tyroch A (2020) Are Concomitant Operations During Bariatric Surgery Safe? An Analysis of the MBSAQIP Database. Obes Surg 30:4474–4481. 10.1007/s11695-020-04848-y 32712783 10.1007/s11695-020-04848-y

[31] Hedberg S, Thorell A, Osterberg J, Peltonen M, Andersson E, Naslund E, Hertel JK, Svanevik M, Stenberg E, Neovius M, Naslund I, Wiren M, Ottosson J, Olbers T, Group BS (2024) Comparison of Sleeve Gastrectomy vs Roux-en-Y Gastric Bypass: A Randomized Clinical Trial. JAMA Netw Open 7:e2353141. 10.1001/jamanetworkopen.2023.53141 38289603 10.1001/jamanetworkopen.2023.53141 PMC10828911

[32] Docimo S Jr., Shah J, Warren G, Ganam S, Sujka J, DuCoin C (2024) A cost comparison of GLP-1 receptor agonists and bariatric surgery: what is the break even point? Surg Endosc 38:6560–6565. 10.1007/s00464-024-11191-1 39285034 10.1007/s00464-024-11191-1

[33] Lauren BN, Lim F, Krikhely A, Taveras EM, Woo Baidal JA, Bellows BK, Hur C (2022) Estimated Cost-effectiveness of Medical Therapy, Sleeve Gastrectomy, and Gastric Bypass in Patients With Severe Obesity and Type 2 Diabetes. JAMA Netw Open 5:e2148317. 10.1001/jamanetworkopen.2021.48317 35157054 10.1001/jamanetworkopen.2021.48317 PMC8845022

[34] Brown WA, Liem R, Al-Sabah S, Anvari M, Boza C, Cohen RV, Ghaferi A, Vage V, Himpens J, Kow L, Morton J, Musella M, Pattou F, Sakran N, Clapp B, Prager G, Shikora S, Collaboration IGR (2024) Metabolic Bariatric Surgery Across the IFSO Chapters: Key Insights on the Baseline Patient Demographics, Procedure Types, and Mortality from the Eighth IFSO Global Registry Report. Obes Surg 34:1764–1777. 10.1007/s11695-024-07196-3 38592648 10.1007/s11695-024-07196-3 PMC11031475

[35] Committee ACI (2012) Updated position statement on sleeve gastrectomy as a bariatric procedure. Surg Obes Relat Dis 8:e21–26. 10.1016/j.soard.2012.02.001 22417852 10.1016/j.soard.2012.02.001

[36] Ozmen F, Sahin TT, Dolgun A, Ozmen MM (2024) Changes in serum ghrelin and resistin levels after sleeve gastrectomy versus one anastomosis gastric bypass: prospective cohort study. Int J Surg 110:5434–5443. 10.1097/JS9.0000000000001608 38833355 10.1097/JS9.0000000000001608 PMC11392113

[37] Olbers T, Lonroth H, Fagevik-Olsen M, Lundell L (2003) Laparoscopic gastric bypass: development of technique, respiratory function, and long-term outcome. Obes Surg 13:364–370. 10.1381/096089203765887679 12841895 10.1381/096089203765887679

[38] Svane MS, Jorgensen NB, Bojsen-Moller KN, Dirksen C, Nielsen S, Kristiansen VB, Torang S, Wewer Albrechtsen NJ, Rehfeld JF, Hartmann B, Madsbad S, Holst JJ (2016) Peptide YY and glucagon-like peptide-1 contribute to decreased food intake after Roux-en-Y gastric bypass surgery. Int J Obes (Lond) 40:1699–1706. 10.1038/ijo.2016.121 27434221 10.1038/ijo.2016.121

[39] English WJ, DeMaria EJ, Hutter MM, Kothari SN, Mattar SG, Brethauer SA, Morton JM (2020) American Society for Metabolic and Bariatric Surgery 2018 estimate of metabolic and bariatric procedures performed in the United States. Surg Obes Relat Dis 16:457–463. 10.1016/j.soard.2019.12.022 32029370 10.1016/j.soard.2019.12.022

[40] Bhandari M, Ponce de Leon-Ballesteros G, Kosta S, Bhandari M, Humes T, Mathur W, Fobi M (2019) Surgery in Patients with Super Obesity: Medium-Term Follow-Up Outcomes at a High-Volume Center. Obes (Silver Spring) 27:1591–1597. 10.1002/oby.22593 10.1002/oby.22593 31479206

[41] Howell RS, Liu HH, Boinpally H, Akerman M, Carruthers E, Brathwaite BM, Petrone P, Brathwaite CEM (2021) Outcomes of bariatric surgery: patients with body mass index 60 or greater. JSLS 25. 10.4293/JSLS.2020.00089 10.4293/JSLS.2020.00089 PMC8241285 34248332

[42] Nasser H, Ivanics T, Leonard-Murali S, Shakaroun D, Genaw J (2019) Perioperative outcomes of laparoscopic Roux-en-Y gastric bypass and sleeve gastrectomy in super-obese and super-super-obese patients: a national database analysis. Surg Obes Relat Dis 15:1696–1703. 10.1016/j.soard.2019.07.026 31530452 10.1016/j.soard.2019.07.026

[43] Wilkinson KH, Helm M, Lak K, Higgins RM, Gould JC, Kindel TL (2019) The Risk of Post-operative Complications in Super-Super Obesity Compared to Super Obesity in Accredited Bariatric Surgery Centers. Obes Surg 29:2964–2971. 10.1007/s11695-019-03942-0 31134478 10.1007/s11695-019-03942-0

[44] Zheng C, Liu Y, Xu C, Zeng S, Wang Q, Guo Y, Li J, Li S, Dong M, Luo X, Wu Q (2024) Association between obesity and the prevalence of dyslipidemia in middle-aged and older people: an observational study. Sci Rep 14:11974. 10.1038/s41598-024-62892-5 38796639 10.1038/s41598-024-62892-5 PMC11127928

[45] Bal J, Ilonzo N, Adediji T, Leitman IM (2021) Gender as a deterministic factor in procedure selection and outcomes in bariatric surgery. JSLS 25 10.4293/JSLS.2020.00077 10.4293/JSLS.2020.00077 PMC7881281 33628005

[46] Alizadeh RF, Li S, Gambhir S, Hinojosa MW, Smith BR, Stamos MJ, Nguyen NT (2019) Laparoscopic Sleeve Gastrectomy or Laparoscopic Gastric Bypass for Patients with Metabolic Syndrome: An MBSAQIP Analysis. Am Surg 85:1108–1112 31657304

[47] Bregion PB, Reis AM, Juca RH, de Oliveira-Filho JR, da Rocha Soares GA, Cazzo E, Ivano VK (2025) Patients with Severe Obesity Undergoing Roux-En-Y Gastric Bypass Versus Sleeve Gastrectomy: A Systematic Review and an Updated Meta-Analysis. Obes Surg 35:1146–1159. 10.1007/s11695-025-07743-6 39964665 10.1007/s11695-025-07743-6

